# Hypochlorous Acid and Chloramines Induce Specific Fragmentation and Cross-Linking of the G1-IGD-G2 Domains of Recombinant Human Aggrecan, and Inhibit ADAMTS1 Activity

**DOI:** 10.3390/antiox12020420

**Published:** 2023-02-08

**Authors:** Yihe Wang, Astrid Hammer, Gerald Hoefler, Ernst Malle, Clare L. Hawkins, Christine Y. Chuang, Michael J. Davies

**Affiliations:** 1 Department of Biomedical Sciences, Panum Institute, University of Copenhagen, 2200 Copenhagen, Denmark; 2 Division of Cell Biology, Histology and Embryology, Gottfried Schatz Research Center, Medical University of Graz, 8010 Graz, Austria; 3 Institute of Pathology, Diagnostic & Research Center for Molecular BioMedicine, Medical University of Graz, 8010 Graz, Austria; 4 Division of Molecular Biology and Biochemistry, Gottfried Schatz Research Center, Medical University of Graz, 8010 Graz, Austria

**Keywords:** aggrecan, ADAMTS, chloramines, extracellular matrix, hypochlorous acid, myeloperoxidase, MPO-H_2_O_2_-Cl^−^ system, protein oxidation

## Abstract

Atherosclerosis is a chronic inflammatory disease and a leading cause of mortality. It is characterized by arterial wall plaques that contain high levels of cholesterol and other lipids and activated leukocytes covered by a fibrous cap of extracellular matrix (ECM). The ECM undergoes remodelling during atherogenesis, with increased expression of aggrecan, a proteoglycan that binds low-density-lipoproteins (LDL). Aggrecan levels are regulated by proteases, including a disintegrin and metalloproteinase with thrombospondin motifs 1 (ADAMTS1). Activated leukocytes release myeloperoxidase (MPO) extracellularly, where it binds to proteins and proteoglycans. Aggrecan may therefore mediate colocalization of MPO and LDL. MPO generates hypochlorous acid (HOCl) and chloramines (RNHCl species, from reaction of HOCl with amines on amino acids and proteins) that damage LDL and proteins, but effects on aggrecan have not been examined. The present study demonstrates that HOCl cleaves truncated (G1-IGD-G2) recombinant human aggrecan at specific sites within the IGD domain, with these being different from those induced by ADAMTS1 which also cleaves within this region. Irreversible protein cross-links are also formed dose-dependently. These effects are limited by the HOCl scavenger methionine. Chloramines including those formed on amino acids, proteins, and ECM materials induce similar damage. HOCl and taurine chloramines inactivate ADAMTS1 consistent with a switch from proteolytic to oxidative aggrecan fragmentation. Evidence is also presented for colocalization of aggrecan and HOCl-generated epitopes in advanced human atherosclerotic plaques. Overall, these data show that HOCl and chloramines can induce specific modifications on aggrecan, and that these effects are distinct from those of ADAMTS1.

## 1. Introduction

Atherosclerosis is a chronic low-grade inflammatory condition and a leading cause of cardiovascular disease globally [1]. This disease is characterized by the formation and growth of plaques in the artery wall, with these characterized by high levels of cholesterol and other lipids, leukocytes (neutrophils, monocytes, and macrophages) and synthetic smooth muscle cells that produce aggrecan, covered by a fibrous cap of extracellular matrix (ECM) and endothelial cells [1,2]. Activation of neutrophils and monocytes results in the release of the heme protein myeloperoxidase (MPO) from storage granules into phagolysosomes and also extracellularly [3]. Release of MPO into the extracellular space results in binding to macromolecules, including plasma proteins, glycoproteins, proteoglycans, and glycosaminoglycans via electrostatic interactions [4].

Reaction of MPO with hydrogen peroxide (H_2_O_2_) and halide (Cl^−^, Br^−^, I^−^) or pseudohalide (SCN^−^) ions generates the corresponding hypohalous acids (hypochlorous acid, HOCl; hypobromous acid, HOBr; hypoiodous acid, HOI; hypothiocyanous acid, HOSCN; review [5]). Under normal physiological conditions, HOCl is a major product due to the high levels of Cl^−^, although other species can act as competing substrates [6]. HOCl is a powerful and highly reactive oxidant that damages most biological molecules, with proteins being major targets due to their high abundance and reactivity [7]. HOCl reacts most rapidly with sulfur-containing amino acid side-chains (cysteine, Cys; methionine, Met; and the disulfide cystine) of proteins and peptides [8,9,10]. Amine groups (e.g., the imidazole side chain of histidine, His; the ε-amino group of lysine, Lys; and the N-terminal amine of proteins) are also significant, though less-reactive targets [8]. Unlike Cys and Met residues, which are present at modest concentrations in the extracellular matrix proteins, amines are abundant in many ECM proteins and likely to be major sites of reaction, due to limited diffusion of HOCl from its site of generation [4]. Reaction of HOCl with amines results in the formation of secondary chloramines [11], which can induce further oxidation as they retain the oxidizing power of hypohalous acid. Both HOCl and chloramines can induce protein cleavage and cross-linking (e.g., [12,13,14]).

Considerable data indicates that high levels of MPO, and its oxidants are associated with tissue damage in both cardiovascular diseases [15,16,17] and other inflammatory pathologies [5,18,19,20]. The steady-state concentrations of HOCl generated in lesions (i.e., the difference between the rates of generation and removal) are difficult to ascertain due to the high reactivity of this species, but previous studies have estimated that concentrations of hundreds of micromolar are generated by activate neutrophils [21,22], and that the total flux to which a protein may be exposed over its biological lifetime may be millimolar [8]. Elevated levels of MPO mRNA, protein, and enzymatic activity are present in human atherosclerotic plaques, together chlorinated (and other) products from reactions of its oxidants [5,23,24,25,26,27]. A strong association has been reported between the extent of MPO-mediated damage and disease severity (e.g., intima-to-media thickness) [28]. Epidemiological data support the hypothesis that high levels of MPO are diagnostic and prognostic of poor health outcomes in people with cardiovascular and other diseases, and also the general population [16,17].

Previous studies have reported that ECM proteins, which MPO can interact with [4] are targets of HOCl-mediated damage. Data have been reported for damage to fibronectin [29] and laminin [30] in human atherosclerotic plaques, as well as the apolipoproteins of low- (LDL) and high-density-lipoproteins [31,32,33,34]. Although the binding sites of MPO on many proteins remain to be established [4], evidence has been presented for MPO binding to the glycosaminoglycan (GAG) chains of the major proteoglycan perlecan [35] and the endothelial cell glycocalyx [36]. Other proteoglycans may also bind MPO and be targets for oxidative damage, arousing its activity. Versican and aggrecan (ACAN), which have similar structures, are potential targets, with both being large chondroitin sulfate (CS)-containing ECM proteoglycans [37,38].

**Figure 1:**
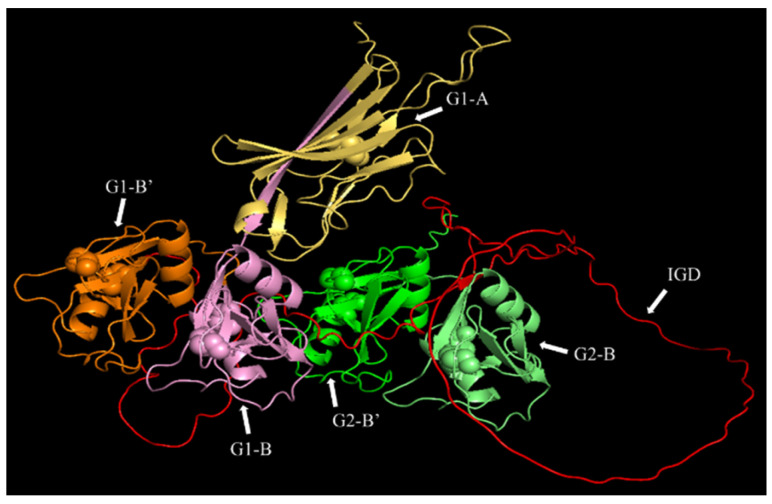
Computational-prediction of the three-dimensional structure of the G1-IGD-G2 domains of recombinant human aggrecan. The G1-A (yellow), G1-B (pink), G1-B’ (orange), interglobular domain (IGD, red), G2-B (light green), and G2-B’ (dark green) domains are indicated. The cysteine-derived disulfide bonds that stabilize the G1 and G2 domains are presented as spheres, with one present in G1-A, and two in each of other globular domains. Structure generated by using AlphaFold v2.2.3 software (London, UK) [48] by using the sequence from the UniProt database (entry P16112) and amino acids Val^20^-Gly^675^.

The core protein of aggrecan is composed of three globular domains (G1, G2, and G3). The G1 domain is responsible for binding to hyaluronic acid and the formation of a hyaluronan-rich ECM [39]. The G1 and G2 domains are separated by a less structured interglobular domain (IGD), whereas the G2 and G3 domains are separated by an extended region which is the site of binding of CS, and lower numbers of smaller keratan sulfate chains [37] (Figure 1). These GAG chains act as binding sites for lipoproteins, and particularly LDL in atherosclerotic plaques [40,41,42,43]. This association is believed to be important in the development of atherosclerosis as it results in increased retention of LDL in the inflamed artery wall [43,44]. This is postulated to allow for LDL oxidation and subsequent uptake by macrophages leading to formation of cholesterol-engorged foam cells [42]. Such oxidation may be enhanced by MPO binding to the proteoglycan, as it would bring the oxidant source and LDL into close proximity. There is, however, little data available on the effects of HOCl on aggrecan, due to the high molecular mass of the intact proteoglycan (1000–2000 kDa, depending on the number of attached sugar chains) and the complexity of this macromolecule.

The IGD between the G1 and G2 domains of aggrecan is susceptible to cleavage, with this being responsible for the loss of aggrecan, release of bioactive (matrikine) fragments, and disruption of cell-ECM interactions [45]. Two principal cleavage sites have been identified: one (the aggrecanase site) between Glu^373^ and Ala^374^, is a target of disintegrin and metalloproteinase with thrombospondin motifs (ADAMTS) enzymes [46], and a second between Asn^341^ and Phe^342^ is a target of matrix metalloproteinases (MMPs) [47]. Other sites have also been reported [46].

In healthy arteries, vascular smooth muscle cells (VSMCs) are present as a contractile, quiescent phenotype. However, they can undergo a phenotypic transition to a synthetic, aggrecan-producing phenotype through interactions with the surrounding ECM [49]. Thus, healthy arteries contain low levels of aggrecan mRNA [43], whereas high levels of aggrecan mRNA and protein are present in both stable and unstable atherosclerotic plaques [50,51]. Aggrecan levels play a key role in determining VSMC behaviour, with elevated concentrations promoting VSMC apoptosis in unstable atherosclerotic plaques [52]. Aggrecan levels are dynamic, and regulated by proteases excreted by VSMCs and other cells. Thus, an increase in mRNA and protein levels of ADAMTS1, 4 and 8 has been observed in response to elevated aggrecan mRNA levels in rats [53]. In contrast, an absence of ADAMTS5 is associated with aggrecan accumulation and worse aneurysmal disease [54,55]. These data indicate that ADAMTS proteases participate in aggrecan turnover to limit accumulation, and are important in disease development. Whether aggrecan degradation is solely proteolytic, or also arises from HOCl-mediated reactions is not known, and the effects of HOCl on ADAMTS activity are unclear. To expand our understanding of these processes, the current study used a truncated recombinant human aggrecan (rhAggrecan) consisting of the G1-IGD-G2 domains with an attached C-terminal His_10_ tag (total molecular mass ~120 kDa), but without the attached GAG chains. Experiments were also carried out with recombinant human ADAMTS1 (rhADAMTS1) that cleaves rhAggrecan at the aggrecanase site. The effects of both HOCl and secondary chloramines present on amino acids, human serum albumin (HSA), specific ECM proteins, and mixtures of these, were examined, together with effects of HOCl and taurine chloramine on ADAMTS1 activity.

## 2. Materials and Methods

### 2.1. Materials

All chemicals including NaCl, HEPES, CaCl_2_, L-methionine (Met), HSA, fibronectin from human plasma (hpFN) and 5,5′-dithiobis (2-nitrobenzoic acid) were from Sigma-Aldrich-Merck (Søborg, Denmark) unless stated otherwise, and all solutions were prepared with Milli-Q grade water (Millipore Advantage A10; Merck-Millipore, Billerica, MA, USA). rhAggrecan (comprising the G1-IGD-G2 domains with a His_10_ C-terminal tag), recombinant human ADAMTS1 (rhADAMTS1) and murine basement membrane extract were from R&D systems (Minneapolis, USA). Murine anti-aggrecan monoclonal antibody (mAb, clone BC3, which recognises a neoepitope with sequence ARGSV arising from cleavage at the Glu^373^-Ala^374^ aggrecanase site), anti-6X His tag antibody (murine mAb, clone number HIS.H8), anti-aggrecan core protein 1 polyclonal antibody (pAb), NuPAGE 4 to 12%, Bis-Tris gels (10- or 12-well, 1.0 mm), NuPAGE MOPS SDS running buffer (20×), NuPage LDS sample buffer (4×) and SuperSignal™ West Pico PLUS Chemiluminescent Substrate were purchased from Thermo Fisher (Roskilde, Denmark). NuPAGE reducing agent (10×) was purchased from Life Technologies (Taastrup, Denmark). Precision Plus Protein Kaleidoscope prestained protein standards (10–250 kDa) were from BioRad (Copenhagen, Denmark). HOCl (10–15% NaOCl, from Acros Organics, Waltham, MA, USA) was quantified by measuring its absorbance at 292 nm by using ε 350 M^−1^ cm^−1^ at pH 11 [56]. *N*α-acetyl-Lys-OH and *N*α-acetyl-His-NHMe (*N*α-acetyl-His with a *N*-methylamide-derivatized carboxyl group) were purchased from Bachem (Bubendorf, Switzerland). Horseradish peroxidase (HRP)-conjugated sheep anti-mouse and HRP-conjugated donkey anti-rabbit pAbs (used as secondary antibodies) were purchased from VWR (Søborg, Denmark). Vivaspin concentrators (molecular mass cut-off: 50 kDa; Sartorius, Germany) was used for buffer exchange for laminin-111. For double-immunofluorescence staining, the murine mAb (clone 2D10G9, raised against HOCl-modified proteins/epitopes [57]) was labelled with a Cy3-Ab-Labelling-Kit according to the manufacturer’s instructions (Cytiva; Traun, Austria).

### 2.2. Formation and Quantification of Chloramines

Next, 4-(2-hydroxyethyl)-1-piperazineethanesulfonic acid (HEPES), amino acid and protein chloramines were prepared as described previously [12,58]. Thus, HEPES-chloramines (HEPES-CA), Lys- (Lys-CA) and taurine-chloramines (Tau-CA) were prepared by adding HOCl to a fivefold molar excess of HEPES, *N*α-acetyl-Lys and taurine, with the parent compounds present at 1 M, 100 mM, and 100 mM respectively, in 5 mM sodium phosphate buffer (pH 7.4). Histidine-chloramines (His-CA) were prepared by adding HOCl to a twofold molar excess of *N*α-acetyl-His-NHMe (40 mM). HSA-chloramines (HSA-CA), human plasma fibronectin-chloramines (FN-CA), laminin-111-chloramines (laminin-CA) and murine basement membrane extract chloramines (BME-CA) were generated by adding a 50-fold molar excess of HOCl over the protein concentration (40 μM HSA, 26 μM fibronectin, 14 μM laminin-111, 20 μM basement membrane extract based on the molecular mass of laminin). Chloramine concentrations were determined by quantifying the oxidation of 5-thio-2-nitrobenzoic acid (TNB) to 5,5′-dithiobis-(2-nitrobenzoic acid) (DTNB) as described previously [12,58]. TNB was generated from DTNB by treatment of 1.3 mM DTNB with 50 mM NaOH for 15 min at 21 °C; this material was then diluted 1:50 in PBS buffer. Samples of the reaction mixture were combined with 1 mL of TNB reagent and incubated for 15 min at 21 °C. The loss of TNB was quantified by its absorbance at 412 nm by using ε 14,150 M^−1^ cm^−1^ [59].

### 2.3. Oxidant Treatments

rhAggrecan (7.4 μL of 200 μg mL^−1^) and/or rhADAMTS1 (3 μL of 56 μg mL^−1^) were mixed with increasing concentrations of HOCl (1- to 777-fold molar excess) or chloramines (10- to 78-fold molar excess) in PBS or assay buffer A (150 mM NaCl, 10 mM HEPES, 5 mM CaCl_2_; pH 7.4) for 24 h at 37 °C). Treatment of rhAggrecan with rhADAMTS1 (6.5:1) in assay buffer A for 24 h at 37 °C was used as a positive control for aggrecan cleavage.

### 2.4. Polyacrylamide Gel Electrophoresis (SDS-PAGE)

SDS-PAGE analysis was carried out under both reducing and nonreducing conditions. Samples (0.62 μg rhAggrecan and/or 70 ng rhADAMTS1) were heated (70 °C, 10 min) in SDS sample buffer (4×) to denature the proteins, and then 20 µL was loaded per well on 4–12% bis-Tris gels (Thermo Fisher) and electrophoresed at 200 V for 50 min. For reducing gels, reducing agent was added prior to loading to a final concentration of 1× from a 10× stock concentration (Life Technologies). Prestained protein standards were used to determine approximate protein masses. Protein bands were visualized by using silver staining as described in [29]. The gels were then scanned, and the band densities analyzed by using ImageJ software (version 1.52a; NIH, Bethesda, MD, USA).

### 2.5. Western Blot Analysis

Proteins were separated by SDS-PAGE (Section 2.4), and then immunoblotted to PVDF membranes by using an iBlot2^®^ Gel Transfer device (Life Technologies). The membranes were then blocked with TBS-T (20 mM Tris-HCl, pH 7.6, 137 mM NaCl, 0.1% v/v Tween 20) containing 1% *w*/*v* bovine serum albumin for 1 h at 25 °C, then probed (4 °C, overnight) with an anti-aggrecan mAb (1:1667) dilution that recognises the ARGSV (aggrecanase cleavage) neoepitope (clone BC3), an anti-His tag mAb (1:3000 dilution), or a pAb against the G1 domain of aggrecan (1:1000 dilution). Epitopes were detected by incubation with HRP-conjugated anti-mouse or anti-rabbit IgG secondary antibodies (1:5000 dilution, 60 min, 21 °C) and developed by using chemiluminescent substrate ECL-detection reagent and imaged by using an Azure imager.

### 2.6. Immunofluorescence Staining of Aggrecan and HOCl-Modified Epitopes/Proteins in Human Atherosclerotic Lesions

Tissue samples (arteriae femuralis) were obtained within 12 h post mortem from three autopsy cases who died from cerebral haemorrhage as described [60,61]. This study was approved by the ethics committee of the Medical University of Graz (EK-number: 29-464 ex 16/17). All procedures were carried out in line with the respective guidelines and regulations. To determine lesion severity, oil red staining was performed as described [60]. After staining the nuclei with hemalum and mounting the sections with Kaiser’s glycerine gelatine, images were obtained by using an Axiophot microscope (Zeiss, Oberkochen, Gemany) with a AxioCam HRc digital camera (Zeiss). Plaque classification was performed as described by Stary et al. [62]. The morphological changes ranged from microscopically normal to slightly thickened intima (lesion type II–III). The samples were frozen in a cryostat (Microm HM500 OM; Microm, Walldorf, Germany) using tissue-freezing medium (Tissue Tec OCT-compound; Miles, Elkhard, IN, USA). Serial cryosections (5 µm thickness) were collected on glass slides, air dried (2 h, 21 °C), fixed in acetone (5 min, 21 °C) and then stored at −80 °C until examination [60]. Before respective analyses, sections were thawed and fixed again in acetone (5 min, 21 °C). Then, sections were rehydrated by using PBS, and blocked with Ultra V block (10 min; Lab Vision, Fremont, CA, USA). For double immunofluorescence staining, sections were first incubated with a mouse anti-aggrecan mAb (clone 969D4D11, IgG1, Invitrogen, Slangerup, Denmark, 1:200 dilution) followed by a goat pAb directed against mouse IgG and labelled with cyanine-2 (Cy-2, Jackson Dianova, Hamburg, Germany, dilution 1:300) as a secondary antibody [63]. After blocking with nonimmune mouse serum (1:25, 15 min), the sections were incubated with Cy-3 labelled mAb 2D10G9 (1:5 dilution) [57]. Dako antibody diluent was used for dilution of the antibodies, and PBS was used for all of the washing steps. Incubations were performed in dark humidified chambers at 21 °C. The sections were mounted with Moviol (Calbiochem-Novabiochem, La Jolla, CA, USA), and analysed by using a confocal laser-scanning microscope operating in sequential mode (Leica TCS SP2, Leica Lasertechnik GmbH, Heidelberg, Germany). Analysis for Cy-2 (green fluoresence) used λ_ex_ 488 nm, and λ_em_ 500–540 nm, and for Cy-3 (red fluoresence) λ_ex_ 543 nm, and λ_em_ 560–620 nm [30]. Control experiments omitted the primary antibodies or replaced them with nonimmune mouse or rabbit IgG (Sigma-Aldrich, Wien, Austria) [30].

### 2.7. Computational Modelling of the Three-Dimensional Structure of the G1-IGD-G2 Domains of Recombinant Human Aggrecan

A computational model of the G1-IGD-G2 domains of recombinant human aggrecan was generated by using AlphaFold v2.2.3 software [48] and the protein sequence from UniProt database entry P16112 with amino acids Val^20^-Gly^675^. The program was executed on a local server running Ubuntu 18.04. The artificial intelligence module “monomer” was used in the modeling, while searching all genetic databases used in CASP14. Finally, the Amber force field of openMM was used to relax the protein model. The model with the highest confidence was selected for use, according to the ordering determined by the software plddt. Pymol software was used to determine the disulfide bond locations.

### 2.8. Statistical Analyses

Data are presented as mean values ± SD, from at least three independent experiments. Gel images are representative examples from three experiments. Integrated density/pixel value of bands on the gel was calculated via ImageJ software (NIH, Bethesda, MD, USA). Statistical analyses were performed by using GraphPad Prism (version 9.0, GraphPad Software, San Diego, CA, USA) by using one-way ANOVA with Dunnett’s multiple comparisons test as indicated in the figure legends. Statistical significance was assumed at the *p* < 0.05 level.

## 3. Results

### 3.1. Effect of HOCl on rhAggrecan

**Figure 2:**
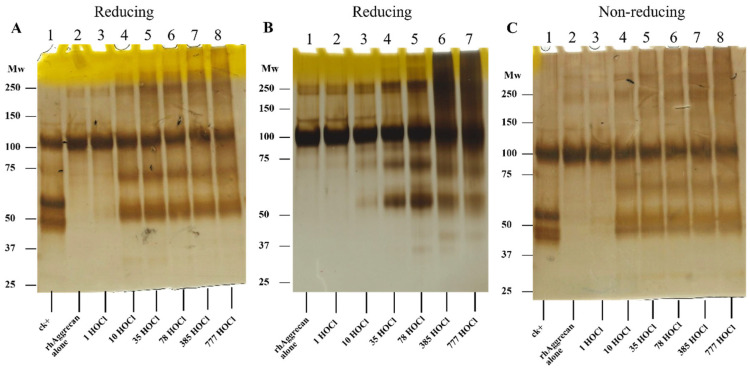
Effects of HOCl on rhAggrecan structure as determined by SDS-PAGE. rhAggrecan (643 nM) was exposed to either 100 nM rhADAMTS1 (positive control, ck+) or 0, 1-, 10-, 35-, 78-, 385- and 777-fold molar excesses of reagent HOCl (i.e., 0, 0.7 μM, 7 μM, 25 μM, 50 μM, 247 μM, and 500 μM) for 24 h at 37 °C in either buffer A (panel **A**,**C**) or in PBS (panel **B**), with the protein then analysed by SDS-PAGE under reducing (panels **A**,**B**) or nonreducing condition (panel **C**), with the protein bands visualized by silver staining. Molecular mass markers are indicated on the left side of each image. Images are representative data from three independent experiments.

In initial experiments, rhAggrecan was treated with increasing concentrations of HOCl (1–777-fold molar excesses) for 24 h at 37 °C (Figure 2A,B). This allowed examination of both the initial (rapid) reactions of HOCl, and effects of long-lived chloramines formed on reaction of HOCl with Lys and His residues. The HOCl-treated protein samples, together with PBS-treated controls and protein treated with ADAMTS1 as a positive control for rhAggrecan cleavage, were subsequently separated by SDS-PAGE under reducing conditions with silver staining. Limited studies were also carried out by using nonreducing conditions. Treatment of rhAggrecan with HOCl in either buffer A (150 mM NaCl, 10 mM HEPES and 5 mM CaCl_2_, pH 7.4; Figure 2A) or PBS (Figure 2B) resulted in changes in protein structure. Buffer A was employed in these experiments as it maintains rhADAMTS1 activity, which is rapidly lost in most buffers. With increasing HOCl concentrations in both buffers, there was an increased detection of material at both higher and lower molecular masses when compared to the untreated, but incubated, parent protein (lane 2, Figure 2A). Material consistent with protein dimers (~240 kDa) was detected at low oxidant excesses (1 to 10-fold, Figure 2A,B), and with greater oxidant excesses, material at higher mass indicating the presence of large aggregates. As these gels were run under reducing conditions, these species are unlikely to involve disulfide cross-links. Limited dimer formation was also observed in the control samples incubated in PBS for 24 h (Figure 2B, lane 1), with less of this detected for the samples in buffer A. Analogous samples in buffer A were also separated by SDS-PAGE on nonreducing gels (Figure 2C). Under these conditions, dimers and aggregates were detected with low levels of HOCl, and these increased at higher oxidant excesses. Comparison of the data from the incubations carried out in buffer A versus PBS indicates that less high-molecular mass material is formed in buffer A, particularly at high HOCl excesses. This may be due to limited reaction of HOCl with the HEPES in buffer A (see also below), or a stabilizing effect of Ca^2+^ on the aggrecan structure.

At similar oxidant excesses to those that gave high mass materials (i.e., 10-fold molar excess of HOCl or greater), two (or more) bands were detected at lower masses (~50 and ~70 kDa) on both reducing and nonreducing gels (Figure 2A–C). As these bands appeared concurrently and showed a similar concentration dependence, they may be generated via a single cleavage of the parent protein backbone, with the combined (apparent) masses similar to that of the parent protein. These fragments are, however, distinct from those obtained from ADAMTS1 cleavage (Figure 2A,C, lane 1 of each image), suggesting that cleavage does not occur at the aggrecanase site.

**Figure 3:**
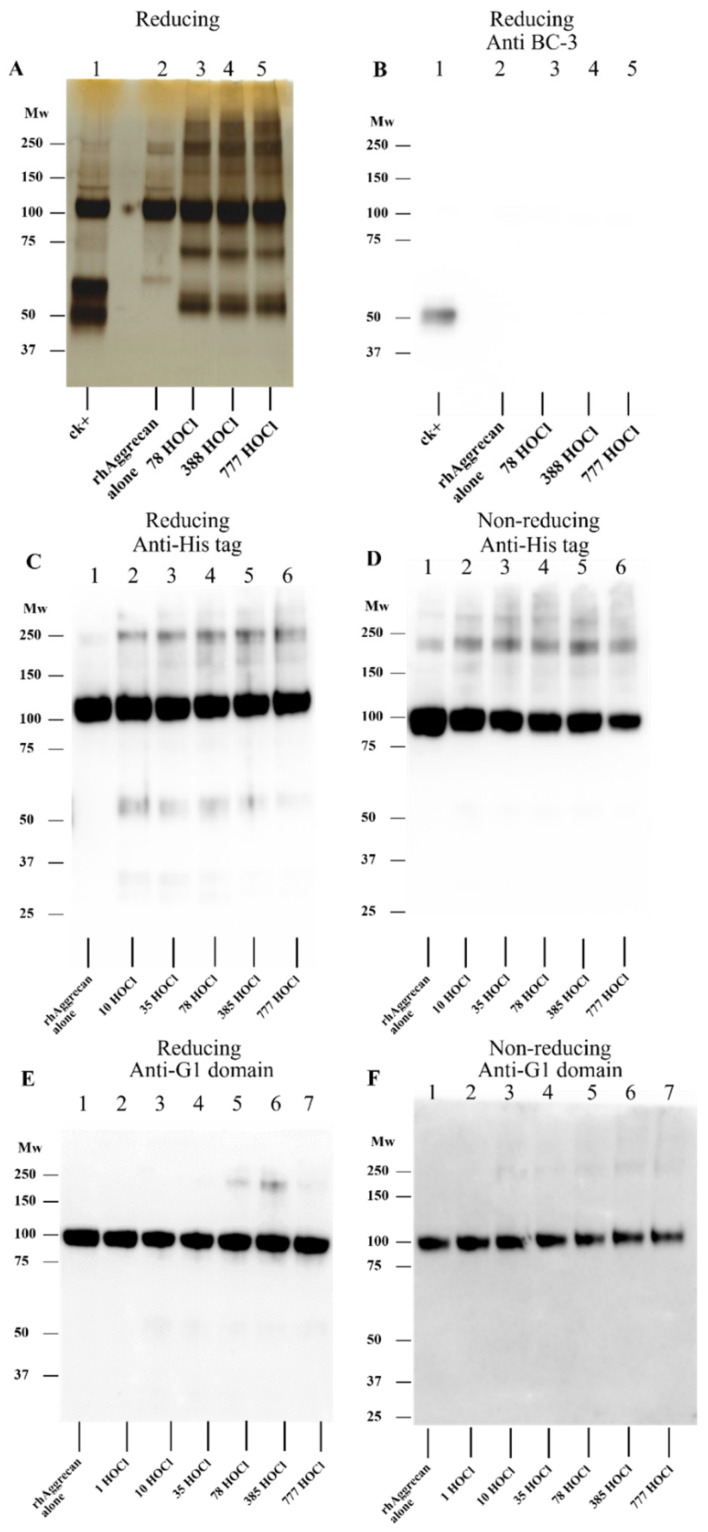
Investigation of HOCl-induced rhAggrecan structural modifications by Western blotting. rhAggrecan (643 nM) was exposed to selected molar excesses of reagent HOCl (0, 78-, 385- and 777-fold) for 24 h at 37 °C in buffer A, with the protein species subsequently separated by SDS-PAGE under either reducing (panels **A**–**C**,**E**) or nonreducing (panels **D**,**F**) conditions. The gels were then either subjected to silver staining (panel **A**, cf. data presented in Figure 2), or blotted to membranes (panels **B**–**E**) and probed by using antibodies. (**B**) Membrane probed by using an antibody that recognizes the neoepitope ARGSV generated by cleavage at the aggrecanase site (mAb BC-3, 1:1667 dilution). (**C**,**D**) Membrane probed by using an antibody that recognizes the His-tag present at the C-terminus (HIS.H8, 1:3000 dilution). (**E**,**F**) Membrane probed by using an antibody that recognizes the G1 (N-terminal) domain of aggrecan (Aggrecan core protein 1 pAb, 1:1000 dilution). Molecular mass markers are indicated on the left side of each image. Representative images from three replicate gels from independent experiments.

Further information on the nature of these fragments was obtained from Western blotting experiments by using antibodies that recognise specific aggrecan epitopes, including an antibody against the neoepitope (ARGSV) generated by cleavage at the aggrecanase site, an antibody against the G1 (N-terminal) domain, and an antibody against the His-tag present at the C-terminus of the G2 domain. Figure 3A presents a silver-stained gel image of rhAggrecan treated with different HOCl molar excesses (as Figure 2A), and Figure 3B shows the corresponding immunoblot after protein transfer to a membrane, and probing with the anti-ARSGV neoepitope antibody (BC-3). As expected, a strong signal was obtained from the positive controls (rhAggrecan treated with rhADAMTS1, lane 1 marked ck+), but no signal was detected from HOCl-treated rhAggrecan, confirming that HOCl does not cleave at the aggrecanase site. Attempts to obtain data on the precise sites of cleavage, by use of in-gel digestion of the fragments and subsequent LC-MS analysis, were unsuccessful due to poor sequence coverage (data not shown).

Analogous membranes were probed by using the His-tag (C-terminus of G2) (Figure 3C,D), and G1 domain (Figure 3E,F) antibodies. The anti-His tag antibody gave a strong signal from the control samples as expected. This band was also detected for the HOCl-treated samples, though the pixel intensity decreased to a limited extent with increasing concentrations of HOCl. Signals were also detected at both lower and higher masses, for gels run under reducing conditions (Figure 3C), with these bands only detected for the oxidant-exposed protein. Under nonreducing conditions (Figure 3D), only the parent protein and higher mass bands were detected. These data indicate that the band at ~50 kDa on the silver-stained gels contains the rhAggrecan C-terminus, and that the dimers also contain this domain as expected. The reason for the absence of lower molecular mass bands on the blots from the gels run under nonreducing conditions is unclear; this may be due to conformational changes which prevent antibody recognition.

For the membranes probed with the anti-G1 (N-terminal domain) antibody, only the parent protein and a weak band from a dimer were detected under reducing and nonreducing conditions (Figure 3E,F). The intensity of the parent band decreased with increasing HOCl excesses, particularly for gels run under nonreducing conditions, suggesting that modifications are formed on the parent protein that decrease antibody recognition. This may also explain the decreased recognition of the dimer observed on reducing gels at high oxidant excesses (Figure 3E).

### 3.2. Effect of Methionine on Oxidation of rhAggrecan by HOCl

**Figure 4:**
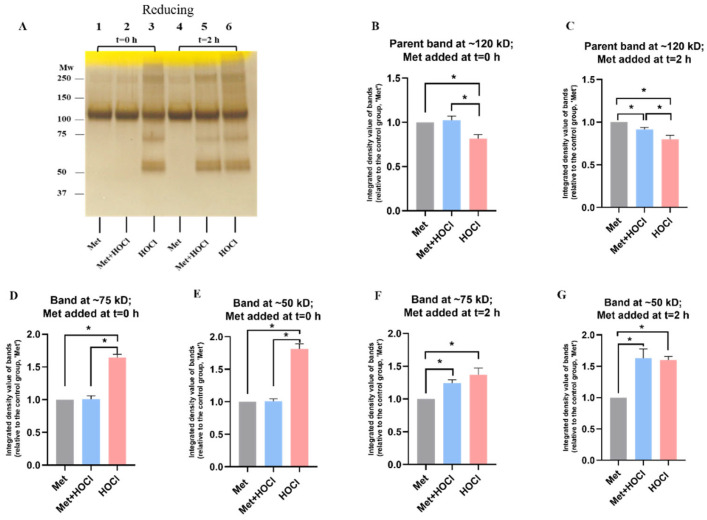
Effects of methionine on HOCl-mediated oxidation on rhAggrecan as detected by SDS-PAGE with subsequent silver staining. (**A**) rhAggrecan (643 nM) was exposed to 78-fold molar excesses of HOCl (i.e., 50 μM) for 24 h at 37 °C in buffer A, in either the absence (PBS added) or presence of Met (1 mM, 20-fold molar excess over HOCl). The Met was added either before the HOCl (t = 0 condition) or after 2 h of reaction. The control and oxidized samples were then subjected to gel electrophoresis under reducing conditions (see Section 2 and legend to Figure 3). (**B**–**G**) The density of the bands from the parent protein and the cleavage fragments (~75 kD and 50 kD) measured as pixel intensities, normalized to the Met + aggrecan control; bar labelled Met) were analysed by using ImageJ software. Statistical analyses were performed by using one-way ANOVA with Tukey’s multiple comparisons test. Statistical differences compared to the Met + aggrecan control (bar labelled Met), the Met + HOCl + aggrecan group (Met + HOCl) and the aggrecan + HOCl (HOCl) are indicated by *, with significance assumed at *p* ˂ 0.05. The gel images are representative data from three independent experiments. Quantitative data obtained from pixel quantification are presented as mean ± SD from three independent experiments.

As the above data indicate that HOCl can modify rhAggrecan, experiments were carried out to determine whether Met (1 mM), which reacts rapidly with HOCl to give an unreactive sulfoxide (second order rate constant, *k*_2_ ~3 × 10^7^ M^−1^ s^−1^ [8]), would prevent protein modification. Met was added either before HOCl (at a 78-fold molar excess), or after 2 h by which time the initial HOCl would be consumed, but chloramines would still be expected to be present and active. Met alone had no effect on rhAggrecan (Figure 4A; lane 1). When Met was added prior to HOCl (t = 0) marked inhibition of fragmentation and cross-linking was observed, and significant protection against loss of the parent protein (Figure 4A, lane 2 versus 3; Figure 4B,D,E). Addition of Met after 2 h had less dramatic effects (Figure 4A, lanes 5 versus 6; Figure 4C,F,G), though a modest decrease in aggregation and fragmentation was observed, consistent with limited effects of chloramines present after 2 h.

### 3.3. Effect of HEPES-Derived Chloramines on rhAggrecan

**Figure 5:**
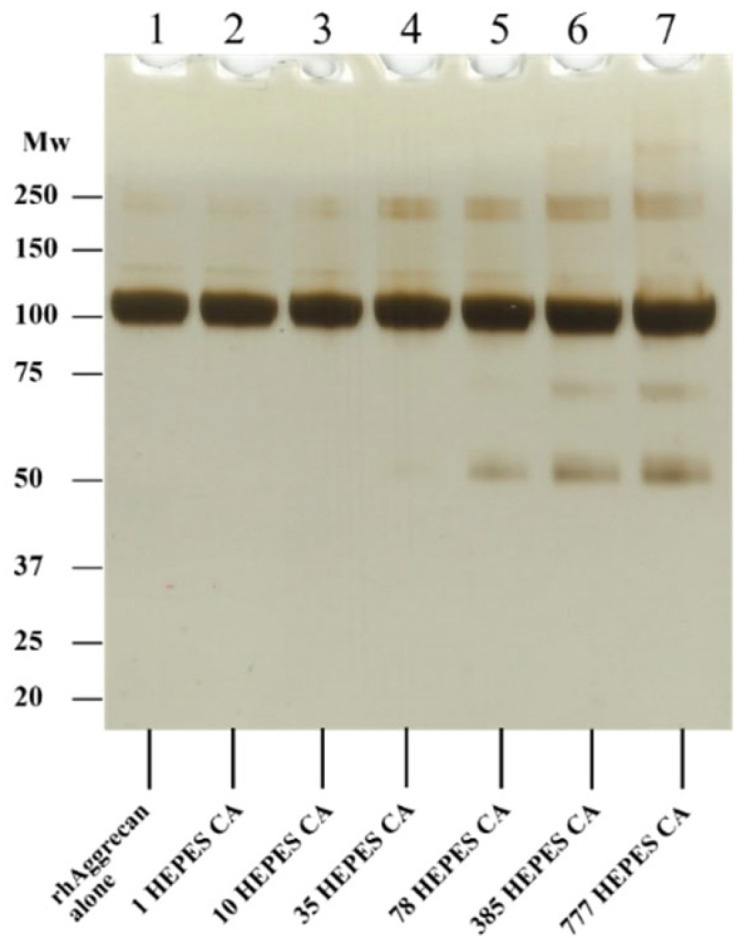
Effects of HEPES chloramines on rhAggrecan. HEPES chloramines (HEPES-CA) were prepared and assayed as described in Section 2 and [58]. rhAggrecan (643 nM) was then incubated with HEPES-CA at molar excesses of 0, 1-, 10-, 35-, 78-, 385-, and 777-fold molar excess over rhAggrecan (i.e., 0, 0.7, 7, 25, 50, 247 and 500 μM) for 24 h at 37 °C in PBS. The samples were then subjected to SDS-PAGE separation under reducing condition, with the protein bands subsequently visualized by silver staining. A representative image from three independent experiments is presented. Molecular mass markers are indicated on the left side of the image.

As buffer A contains HEPES, which might yield chloramines (cf. [64], but also [58], which suggests that this may be limited), HEPES chloramines were generated deliberately [58] and incubated with rhAggrecan at similar molar excesses to those used in the HOCl experiments, to examine differences between the buffers (see Section 3.1) in more detail. These experiments (Figure 5) indicate that high molar excesses of HEPES chloramines are required to generate significant cross-links and fragmention of rhAggrecan. Cross-links were detected only with 35- or greater molar excesses, and 78-fold molar excess of HEPES chloramines were required to generate fragments. These data suggest that HEPES chloramines, if formed in buffer A, do not markedly interfere (c.f. [58]), though HEPES may scavenge some HOCl when this is present at high concentrations.

### 3.4. Effect of Amino Acid-Derived Chloramines on rhAggrecan

As chloramines may be formed in abundance in biological fluids due to the levels of free amino acids and proteins in plasma and the ECM, and some of these have high reactivity [7,65], studies were carried out with both low molecular mass (taurine, Tau-CA; *Nα*-acetyl-lysine, Lys-CA; and *Nα*-acetyl-His-NHMe, His-CA) and protein-derived (HSA) chloramines. The formation of these species and their concentrations was assessed by using TNB (see Section 2).

**Figure 6:**
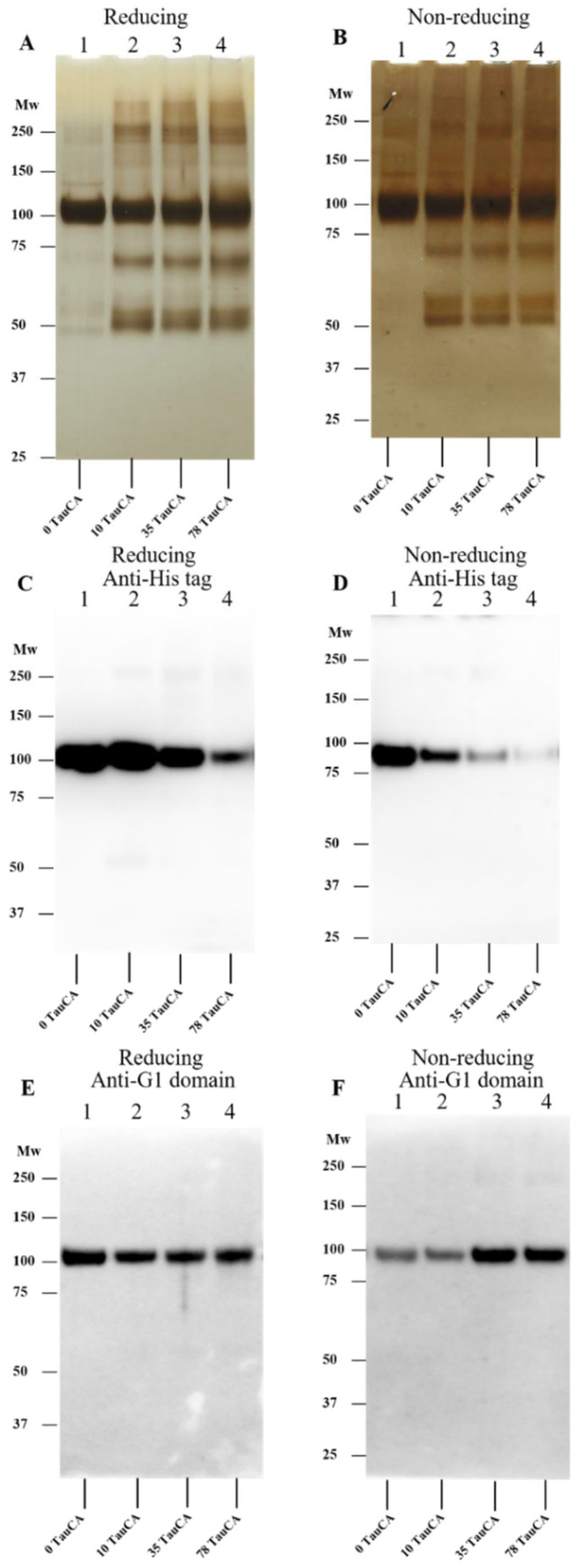
Effects of different concentrations of TauCA on rhAggrecan. Preformed chloramines (see Section 2) were incubated, at the indicated molar excesses with rhAggrecan (643 nM) for 24 h at 37 °C in buffer A, before examination by SDS-PAGE with subsequent silver staining (panel **A**, reducing conditions; panel **B**: nonreducing condition) and Western blotting (panels **C**–**F**) by using an anti-His-tag (C-terminus) antibody (panels **C**,**D**: reducing and nonreducing gels, respectively) and an anti-G1 domain antibody (panel **E**: reducing, panel **F**: nonreducing). For further details, see Section 2 and the legend to Figure 3. Representative images from three independent experiments are presented. Molecular mass markers are indicated on the left of each image.

**Figure 7:**
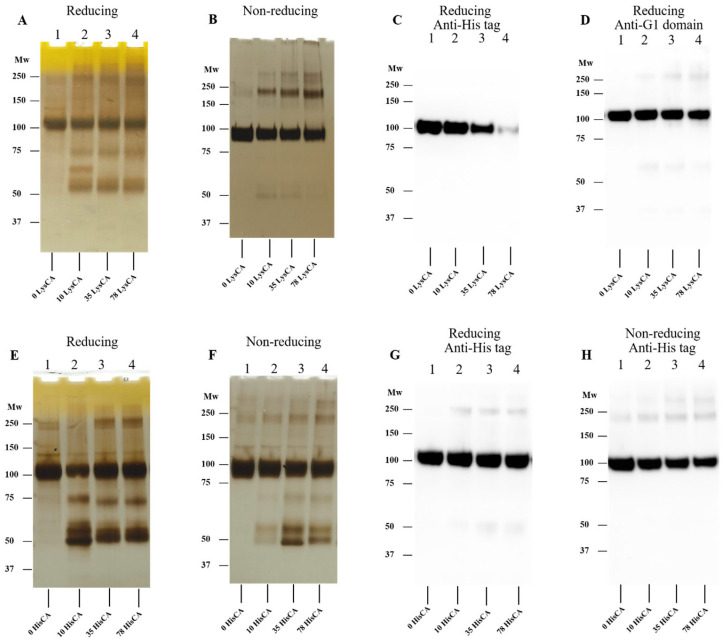
Effects of Lys-CA (panels **A**–**D**) and His-CA (panels **E**–**H**) on rhAggrecan. Preformed chloramines (see Section 2) were incubated with rhAggrecan (643 nM) in buffer A for 24 h at 37 °C before examination by SDS-PAGE with subsequent silver staining (panels **A**,**C**–**E**,**G**, reducing gels, panel **B**,**F**,**H**, nonreducing conditions), or immunoblotting by using an anti-His-tag antibody (panel **C**,**G**,**H**) or an anti-G1 domain antibody (panels **D**). For further details, see Section 2 and the legend to Figure 3. Representative images are presented from three independent experiments. Molecular mass markers are indicated to the left of each image.

In each case, rhAggrecan was treated with 10-, 35-, and 78-fold molar excesses of pre-formed chloramines (i.e., 6.4, 22.4 and 50 μM) with the samples, and controls, subsequently examined by SDS-PAGE with silver staining, and in some cases Western blotting by using the anti-His tag mAb and anti-G1 domain pAb. The resulting data (Figure 6 and Figure 7) indicate that Tau-CA, Lys-CA and His-CA can induce fragmentation and dimer formation from rhAggrecan in a manner similar to HOCl, though to varying extents. Three fragment bands (at ~50, ~60, and ~70 kD) were apparent when rhAggrecan was treated with Tau-CA (with this especially evident on nonreducing gels; see Figure 6B), and this was also evident with low molar excesses of Lys-CA (Figure 7A) and His-CA (Figure 7D,E). With His-CA, fragment formation was maximal at modest oxidant excesses, and become less distinct at higher concentrations (Figure 7D,E). Dimer formation only occurred to a significant extent at higher oxidant excesses (78-fold) than with Tau-CA and Lys-CA. With the latter two chloramines both processes were detected with 10-fold molar excesses of oxidant. In analogous Western blot experiments, much greater epitope loss was detected with the anti-His tag (C-terminal) antibody than the anti-G1 domain antibody (Figure 6 and Figure 7), though some loss of G1 recognition was detected at high oxidant excesses (e.g., for Tau-CA on reducing, Figure 6E, but not nonreducing gels Figure 6F). The greater sensitivity to epitope loss for the His-tag antibody may reflect damage to the G2 domain (to which the His-tag is attached) or alternatively modification of the His-tag. The loss of anti-His tag recognition was more marked with chloramines than HOCl (Figure 3C,D), and was more dramatic with Lys-CA (Figure 7B) than Tau-CA (Figure 6C) under identical conditions. This difference may reflect the greater reactivity of Lys-CA compared to Tau-CA [7,65].

### 3.5. Effect of Human Serum Albumin-Derived Chloramines on rhAggrecan

**Figure 8:**
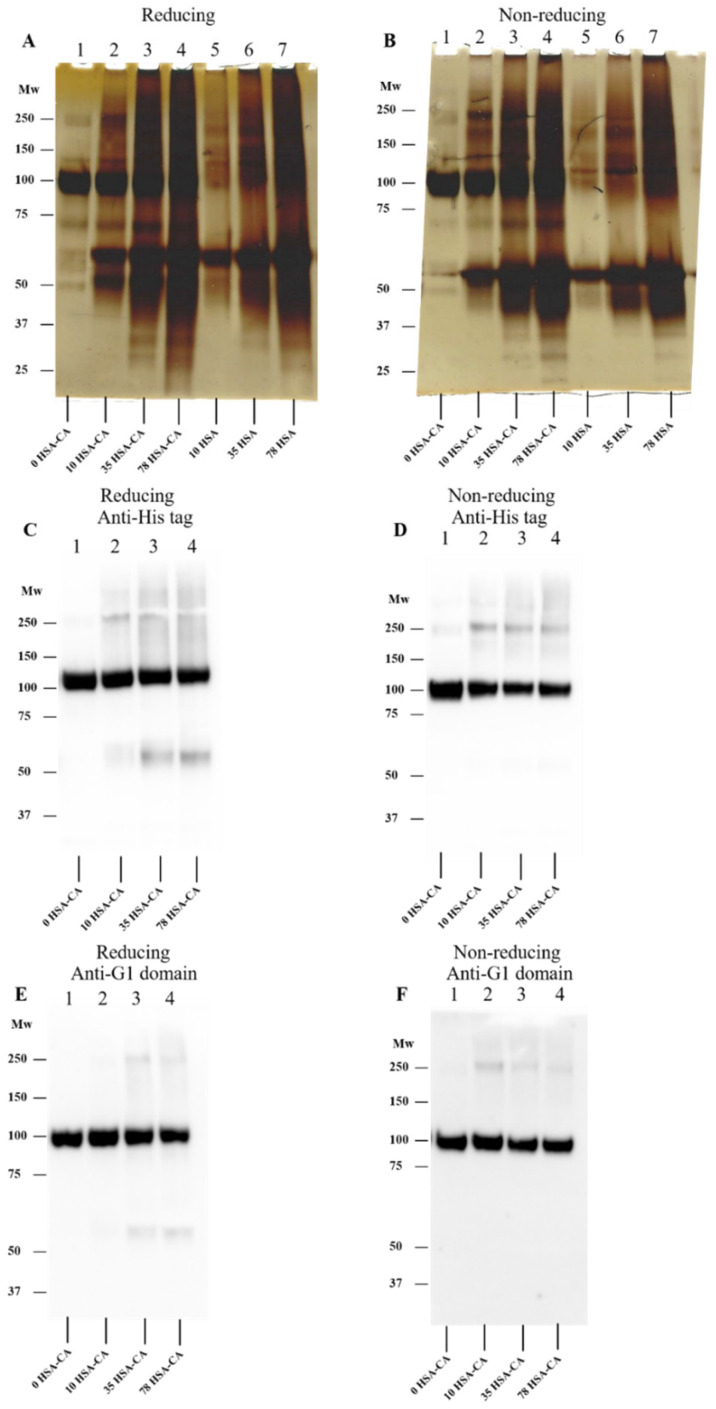
Effects of HSA chloramines (HSA-CA) on rhAggrecan. Defined molar excesses of HSA-CA were incubated with rhAggrecan (643 nM) for 24 h at 37 °C in buffer A. Control samples containing PBS-treated HSA at identical HSA concentrations were also run (bars labelled 10 HSA-control, etc.) before analysis by SDS-PAGE with silver staining (**A**,**B**) or Western blotting by using the anti-His antibody (**C**,**D**), or the anti-G1 domain antibody (**E**,**F**), with the gels run under reducing condition (**A**,**C**,**E**) or nonreducing condition (**B**,**D**,**F**). For further details see Section 2 and the legend to Figure 3. The position of molecular mass markers is indicated on the left of each image. Representative images are presented from three independent experiments.

rhAggrecan was incubated with 10- to 78-fold molar excesses of HSA-CA before analysis as reported above. The data obtained (Figure 8) indicate that HSA-CA can induce rhAggrecan fragmentation and aggregation at all the chloramine concentrations tested. As analysis of the SDS-PAGE gels (Figure 8A,B) is complicated by additional bands from HSA (monomer, dimer, aggregates), control samples containing identical concentrations of PBS-treated HSA (labelled 10-, 35- and 78-HSA control etc, with the numberical value referring to the molar excesses) were included on all gels. Despite this complexity, additional bands were detected in the chloramine-treated samples compared to the controls, with rhAggrecan fragments detected at ~70 kDa and ~50 kDa, as seen with the low molecular mass chloramines. The additional fragment detected with some of the other chloramines at ~60 kDa may also be present but obscured by the parent HSA band. rhAggrecan dimers and aggregates were also detected, with greater staining detected above the parent aggrecan band (e.g., Figure 8A,B, lanes 3 versus 6 in each case), though this was partly confounded by HSA aggregate bands. Confirmation of rhAggrecan dimer and aggregate formation was obtained from Western blot experiments (from both reducing and nonreducing gels, Figure 8C–F), where confounding HSA bands were not detected.

### 3.6. Effect of ECM Protein-Derived Chloramines on rhAggrecan

**Figure 9:**
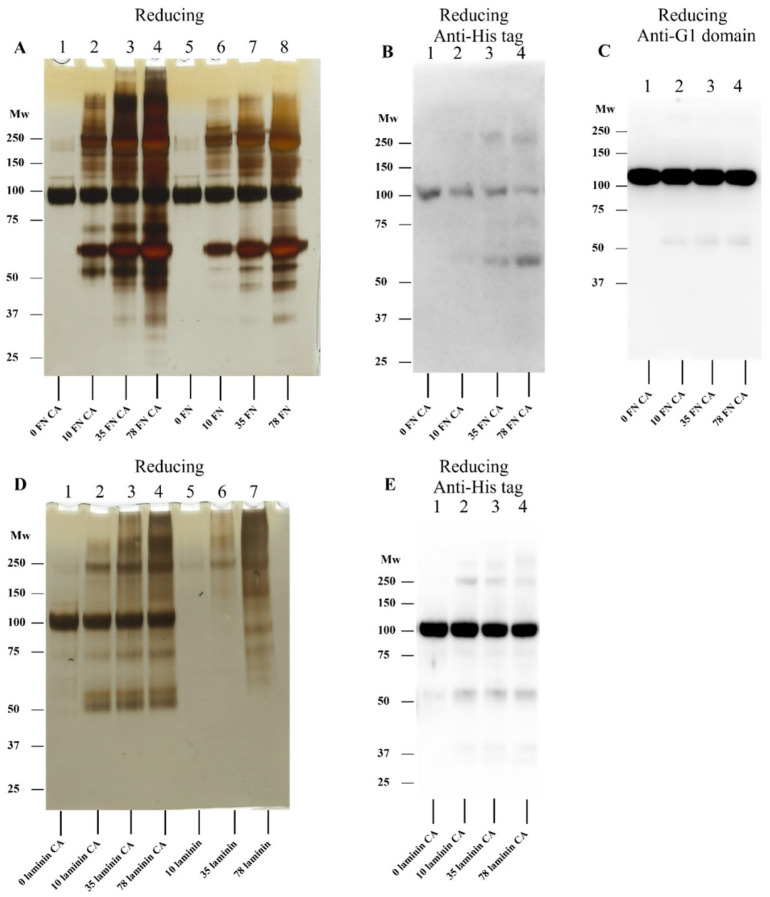
Effects of human plasma fibronectin (FN-CA) and laminin (laminin-CA) chloramines on rhAggrecan. Defined molar excesses of preformed chloramines (as indicated; bars labelled FN CA and laminin-CA), together with identical concentrations of the proteins treated with PBS in place of HOCl (lanes marked FN control and laminin control), were incubated with rhAggrecan (643 nM) for 24 h at 37 °C in buffer A. The samples were then subjected to gel electrophoresis under reducing conditions (panel **A–C** for FN, panels **D,E** for laminin, respectively) and Western blotting by using the anti-His-tag antibody (panels **B**,**D,E**) and anti-G1 domain (panel **C**) as described in Section 2 and the legend to Figure 3. Molecular mass markers are indicated on the left of each image. Images are representative data from three independent experiments.

**Figure 10:**
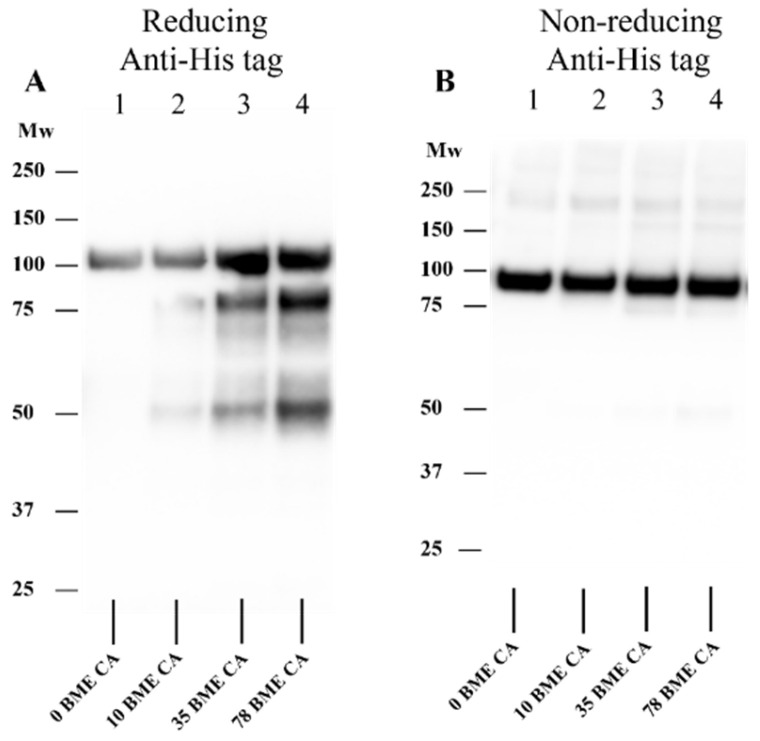
Effects of basement membrane extract chloramines on rhAggrecan. Chloramines were generated on murine basement membrane extract (BME, 20 µM) exposed to a 50-fold molar excess of HOCl (1 mM). Defined concentration of the preformed chloramines (as indicated; bars labelled BME-CA) was then incubated with rhAggrecan (643 nM) for 24 h at 37 °C in buffer A. The samples were then subjected to gel electrophoresis under reducing (**A**) or nonreducing (**B**) conditions and transfer to membranes before Western blotting with the membranes probed with the anti-His-tag antibody. Molecular mass markers are indicated to the left of each image. Images are representative data from three independent experiments.

Studies were also undertaken with chloramines generated on two abundant ECM proteins (human plasma fibronectin, hpFN; and laminins), as well as ECM proteins present in a basement membrane extract. The resulting data (Figure 9 and Figure 10) indicate that chloramines present on hpFN (FN-CA), laminin-111 (laminin-CA), and murine basement membrane extract proteins (BME-CA) can induce fragmentation and dimerization/aggregation of rhAggrecan over a range of molar excesses (10-, 35- and 78-fold molar excess over rhAggrecan; 6.4, 22.4 and 50 μM chloramines, respectively). Although parent hpFN and laminin gave rise to multiple additional bands on the gels, rhAggrecan fragments (~70 and ~50 kDa), and dimers/higher mass aggregates were detected on chloramine treatment, with these not detected in controls (hpFN and laminin treated with PBS in place of HOCl; Figure 9A,D). The formation of these species increased in a dose-dependent manner with the chloramine molar excess. Western blotting experiments confirmed the presence of fragments and dimers (Figure 9B,C,E). For the basement membrane extract experiments, the SDS-PAGE gels did not provide analyzable data, due to the large number of proteins in the samples, with near-continuous smears detected on the gels. However, Western blotting experiments with the anti-His tag antibody provided evidence for rhAggrecan fragments, and limited dimer generation (Figure 10A,B). Analogous experiments were not carried out with the anti-G1 domain antibody due to the presence of aggrecan in the basement membrane extract preparations, which resulted in strong background signals.

### 3.7. Effect of HOCl on Isolated rhADAMTS1 Activity

**Figure 11:**
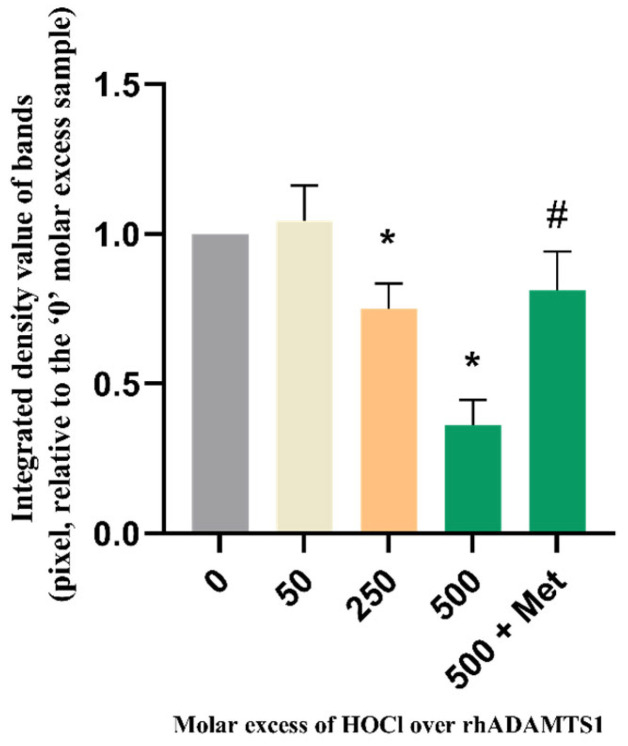
Effect of HOCl on rhADAMTS1 cleavage activity. rhADAMTS1 (99.8 nM) was exposed to 0, 50-, 250- and 500-fold molar excesses of reagent HOCl (0, 5 μM, 25 μM, and 50 μM, respectively) for 15 min at 37 °C, before addition of rhAggrecan (molar ratio of rhAggrecan:rhADAMTS1, 6.5:1) and incubation for a total of 24 h at 37 °C. Samples were separated by SDS-PAGE, transferred to membranes and subjected to Western blotting by using the BC-3 mAb (1:1667 dilution) as indicated in the legend to Figure 3. The pixel density of the band (determined by using ImageJ software) detected for the untreated enzyme was compared to the HOCl-treated samples. In some experiments with a 500-fold molar excess of HOCl, 1 mM Met was added before addition of the HOCl. Data are presented as mean ± SD from three independent experiments. Statistical analysis was performed relative to the 0-fold molar excess of oxidant by using one-way ANOVA with Dunnett’s multiple comparisons test (for the different HOCl concentrations) and unpaired t test with Welch’s correction (for the effect of Met). Statistical significance (*p* < 0.05) compared to the untreated enzyme is indicated by *; significance of the Met treatment, compared to the 500-fold molar excess of HOCl, is indicated by #.

As HOCl and other oxidants can both activate and inactivate MMPs [58,66,67,68,69,70], it was of interest to determine whether this also occurred with ADAMTS1. rhADAMTS1 was treated with HOCl (0–500-fold molar excesses over enzyme) for 15 min at 37 °C, then samples of the treated and native enzyme were added to rhAggrecan and further incubated for 24 h at 37 °C in buffer A, with generation of the neoepitope (ARGSV) from rhADAMTS1-mediated cleavage at the aggrecanase site detected using the BC-3 mAb (cf. Figure 3B). The intensity of the neoepitope band was then expressed relative to that generated by ADAMTS1 not exposed to HOCl. Samples were also analyzed in which Met (1 mM) was included before HOCl addition. These data (Figure 11) indicate that no activation of ADAMTS1 occurs (i.e., pixel intensity greater than controls), with only inactivation detected at all HOCl concentrations tested; this was significant at molar excesses > 250-fold. Loss of activity was almost completely prevented by Met (1 mM) at the highest HOCl concentration examined.

### 3.8. Effect of HOCl on rhADAMTS1 Cleavage of rhAggrecan

**Figure 12:**
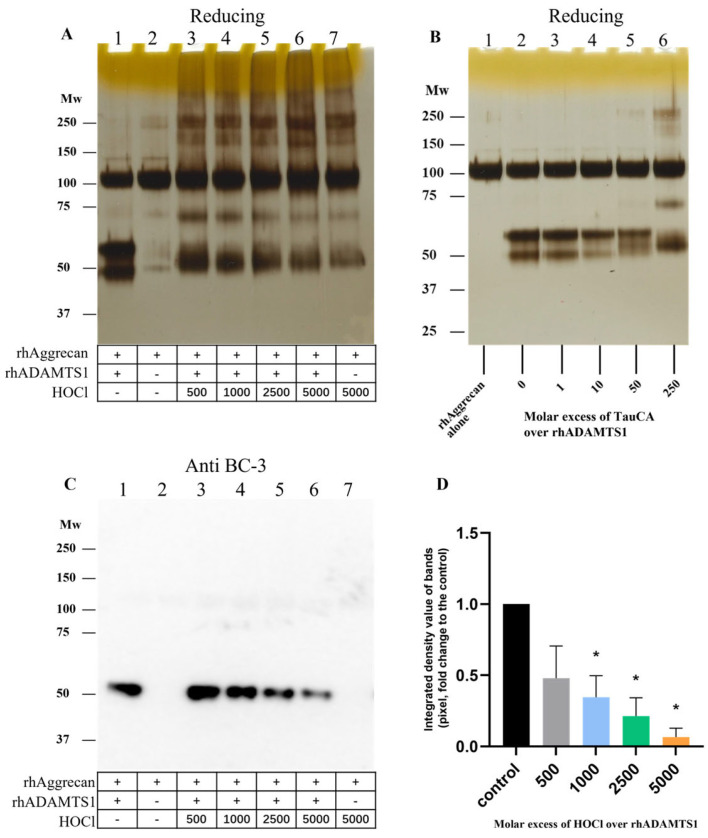
Effects of HOCl and TauCA on rhADAMTS1-mediated cleavage of rhAggrecan. (**A**,**C**) rhAggrecan was incubated with rhADAMTS1 in the absence and presence of HOCl at molar ratios of 6.5:1:500–5000 for 24 h at 37 °C, with subsequent analysis by SDS-PAGE (panel **A**) and Western blotting by using the BC-3 mAb (1:1667 dilution) against the neoepitope fragment generated by ADAMTS1-mediated cleavage of aggrecan (panel **C**). Panel **D**: Densitometric analysis of the pixel density of the bands from panel C determined by using ImageJ relative to the control (no added HOCl; lane 1, panel **C**). Panel B: rhADAMTS1 (100 nM) was exposed to 0, 1-, 10-, 50- and 250-fold molar excesses of TauCA (i.e., 0, 0.1 μM, 1 μM, 5 μM and 25 μM) for 15 min at 37 °C, before addition to rhAggrecan (643 nM) and further incubation for a total of 24 h at 37 °C. The control (panel **B**, lane 1) and oxidized samples were then subjected to SDS-PAGE as described in Section 2 with subsequent silver staining. Images are representative data, and quantitative data are mean values ± SD, each from three independent experiments. Statistical analysis was performed by using one-way ANOVA with Dunnett’s multiple comparisons test with statistical significance compared to the control (0 µM HOCl) indicated by *, with significance assumed at *p* < 0.05.

As both rhADAMTS1 and HOCl can cleave rhAggrecan, and HOCl can inactivate rhADAMTS1 (see above), it was of interest to determine whether HOCl also inhibited ADAMTS1 in the presence of rhAggrecan, and what fragments might be formed when all three components were present simultaneously, as is likely to occur in vivo. rhADAMTS1, rhAggrecan and HOCl (molar ratios: 1:6.5:500–5000) were incubated for 24 h at 37 °C with fragment and aggregate formation examined by SDS-PAGE and Western blotting by using the BC-3 mAb to detect the neoepitope generated by rhADAMTS1 cleavage. In the absence of HOCl, the two expected fragments from enzymatic cleavage were detected (Figure 12A, lane 1 versus lane 2). In the additional presence of increasing concentrations of HOCl, these bands were lost, and the fragments and dimer/aggregates generated by HOCl were detected instead (Figure 12A, lanes 3–7). The corresponding Western blot for the rhADAMTS1 neoepitope confirmed a decreased yield of this fragment with increasing HOCl exposure (Figure 12C,D), with this ascribed to HOCl-mediated rhADAMTS1 inactivation.

### 3.9. Effect of TauCA on rhADAMTS1-Mediated Cleavage of rhAggrecan

To determine whether chloramines also modulate rhADAMTS1 activity, experiments were carried out with Tau-CA at 0–250-fold molar excesses for 15 min (Figure 12B), before addition to rhAggrecan and incubation for a total of 24 h at 37 °C. Under these conditions, only modest effects of Tau-CA were detected on rhADAMTS1 cleavage of rhAggrecan. Thus, the two fragments arising from enzymatic cleavage at the aggrecanase site were detected with low concentrations of Tau-CA, with these decreasing in intensity with increasing Tau-CA concentrations. Only at the highest oxidant doses were the fragment and dimer bands arising from oxidant damage detected (cf. Figure 6). These data indicate that Tau-CA is less efficient at inhibiting rhADAMTS1 than HOCl.

### 3.10. Evidence for HOCl-Mediated Aggrecan Damage in Advanced Human Atherosclereotic Plaques

**Figure 13:**
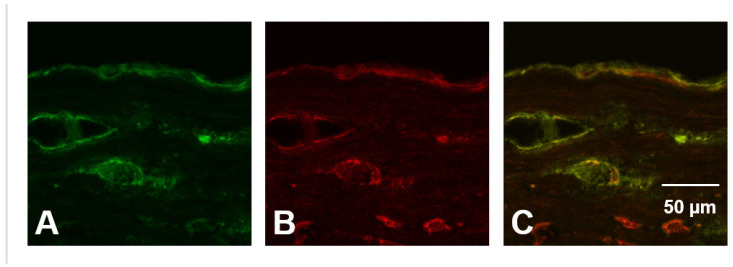
Immunofluorescence staining for aggrecan and HOCl-modified epitopes and colocalization in human atherosclerotic lesion material. Frozen sections (5 μm thick) of an arteria femuralis from a 56-year-old female patient (lesion type II-III) were incubated with a mAb against aggrecan followed by an incubation with a Cy-2 (green fluorescence)-labelled goat anti-mouse IgG used as a secondary antibody (panels **A**,**C**). HOCl-modified proteins were detected by using a Cy-3-labelled mAb (clone 2D10G9, red fluorescence, **B**,**C**). Merged images (**C**; yellow fluorescence) show the resulting overlay of aggrecan and HOCl-modified epitopes, with a significant co-localization detected in the basement membrane underlying the endothelial cell layer (top part of image **C**) and around vasa vasorum. Cells (most likely macrophages as deduced from morphology) located in the lower part of the image do not react with the anti-aggrecan mAb but show a strong signal for HOCl-modified proteins. Oil red staining revealed disperse cholesterol deposits in the intima and media of the sclerotic arteries and macrophages (data not shown). Scale bar: 50 μm.

To examine potential HOCl-mediated damage to aggrecan in vivo possible colocalization of aggrecan and HOCl-generated epitopes was examined. Human atherosclerotic lesions (Types II-III as determined by using the Stary classification [62] and checked by oil-red staining) were cryosectioned, and then incubated with either a mAb specific for aggrecan (clone 969D4D11) or mAb 2D10G9 (which recognizes HOCl-generated epitopes [57]) before analysis by confocal microscopy. Significant fluorescence intensity (green, Cy-2) from the mAb recognizing aggrecan was detected in the plaques, with this localized in the intima, and particularly the basement membrane underlying the endothelial cell layer, as well as the regions surrounding the vaso vasorum (Figure 13A,C). The Cy-3 labelled 2D10G9 mAb gave significant (red) fluorescence from similar regions of the arterial wall (Figure 13B,C). In addition, cells exhibiting the morphology and localization of macrophages in atherosclerotic plaques showed a strong signal for HOCl-modified proteins; this fluorescence may arise, in part, from HOCl-modified LDL which is recognized by this antibody. These cells did not react with the mAb against aggrecan confirming specificity of staining. Control experiments carried out in the absence of the primary antibodies did not give rise to significant fluorescence. Merging of these pairs of images (Figure 13C, yellow fluorescence) provided data consistent with the colocalization of aggrecan and HOCl-generated epitopes in the basement membrane and vasa vasorum. The additional areas of red fluorescence in these images suggest that other targets for HOCl are also present in these samples. Corresponding studies with healthy tissue were not undertaken due to the poor availability of such material, and previous reports indicating that little or no aggrecan is present in healthy aortic tissue (although markedly elevated levels are present in diseased specimens [43]).

## 4. Discussion

Considerable data links high levels of MPO, and its oxidants with cardiovascular diseases [15,16,17] and other inflammatory pathologies [5,18,19,20]. MPO binds to ECM materials, and particularly negatively charged structures including glycoproteins, glycosaminoglycans, and proteoglycans in human atherosclerotic plaques [29,30]. Aggrecan, which is abundant in developing plaques [43,44] but present at low levels, or absent from, healthy aortic tissues [43], would therefore be expected to be both a binding site for MPO, and a target for HOCl damage. Aggrecan is also expected to bind LDL (cf. data for versican [40,41,42,43]), so this species may act as a “scaffold” that localizes MPO and LDL, thereby enhancing LDL oxidation, an event strongly associated with the development of lipid-laden (foam) cells and plaques [32,33,34,42]. Despite these links, little previous work has been carried out on the reactions of HOCl with aggrecan, probably reflecting the large and complex nature of this proteoglycan. Although the current studies have used a truncated version of the protein, consisting of the G1, IGD and G2 domains of the full-length protein (which also contains the chondroitin sulfate binding sequence and G3 domain), this form may be of biological relevance and importance. Thus, data has been presented for a decrease in the levels of full-length protein in aortic tissue from older donors, as evidenced by a marked reduction in recognition of material by antibodies against the chondroitin sulfate binding sequence and G3 domains [71]. In contrast, significant recognition of the G1-G2 domain has been detected in aged tissue samples, together with the formation of fragmented aggrecan species [71]. The presence of the chondroitin chains in the full-length protein may influence the observed chemistry, as they may shield the core protein from damage induced by HOCl. However, as these chains may act as a binding site for MPO, they may also have the opposite effect (i.e., enhance damage, as observed with the related proteoglycan perlecan [35]). These possibilities are worthy of further study.

The current study has provided evidence for HOCl-mediated damage to the G1-IGD-G2 domains of rhAggrecan, with this occurring in a dose-dependent manner. Nonreducible dimers and higher aggregates (probably tetramers; Figure 2 and Figure 3A) have been detected, together with specific fragments. Chloramines generated similar species, with this occuring not only with low-mass species (chloramines formed on taurine, lysine, and histidine), but also those present on HSA, isolated fibronectin, and laminin, and the mixtures of proteins present in a basement membrane extract. These data indicate that efficient damage transfer can occur from other amino acids and proteins present in human atherosclerotic plaques to aggrecan.

Previous studies on the reactions of HOCl with isolated proteins (e.g., HSA [12], fibronectin [29], laminins [30]), protein mixtures (e.g., human plasma [13]), and lipoproteins [25,31,72,73], have provided evidence for protein cross-linking / aggregation. Some of these cross-links have been identified (on the basis of their loss under reducing conditions) as interchain disulfides (e.g., [74]). Others are nonreducible, including di-tyrosine (e.g., [75]), tryptophan-derived linkages (e.g., [76]) or Schiff-based structures [72]. Sulfenamide, sulfinamide, and sulfonamide links have also been identified [77,78]. In other cases, aggregation appears to arise from noncovalent interactions (e.g., with apomyoglobin and apohemoglobin [79]). Although this has not been investigated here, some of the above linkages may underlie the observed dimerization and aggregation of aggrecan.

The chemistry of HOCl-mediated protein fragmentation is less well established. In many cases, fragmentation has been detected as a “smearing” consistent with the generation of many species of different masses, rather than well-defined fragments of specific mass. Such smearing may be due, at least in part, to direct and nonspecific reaction of HOCl with backbone amides and subsequent hydrolysis, though it may also arise from decomposition of chloramines/chloramides to radicals, and radical-mediated backbone cleavage [12,13,14].

In contrast to these nonspecific reactions, HOCl and chloramines gave rise to a small number of well-defined fragments from rhAggrecan. One of these has a molecular mass of ~70 kDa, and the other ~50 kDa. Whether the staining at ~50 kDa is from a single or multiple species is not clear. Gels run under nonreducing conditions (Figure 2C) appear to show two species, but samples run under reducing conditions gave only a broad band at this mass (e.g., Figure 2A and Figure 3A). The two bands at ~50 kDa observed under nonreducing conditions may reflect two conformations of the same species (e.g., with and without a cleaved disulfide bond), with this distinction removed on reduction. The exact masses of these fragments cannot be determined, as gel migration is not only dependent on mass, but also conformation, charge, and SDS binding. However, it is likely that the fragment(s) have masses of approximately these values. Consideration of the protein sequence indicates that cleavage is likely to occur within the IGD domain, if it is assumed that these species arise from a single cleavage event, rather than multiple events, which are statistically unlikely to give defined species. The observation of these bands, at apparently identical masses across the different oxidant systems, suggests a common pathway and site of cleavage.

The fragments generated by HOCl and chloramines are distinct from those generated by ADAMTS1, which cleaves between Glu^373^ and Ala^374^ [47], with the observed bands having different masses (Figure 2 and Figure 3A,B). In addition, Western blotting using the BC-3 antibody which recognizes the neoepitope generated by ADAMTS1, recognized only one enzymatically-generated fragment (at ~50 kDa, as expected), but none of the fragments formed by HOCl (Figure 3B). In contrast, Western blotting with the anti-His tag antibody recognized the ~50 kDa species generated by HOCl, confirming that this species contains the C-terminus of the protein. The dimer and higher aggregates formed by HOCl also stained positively with this antibody, and the anti-G1 domain antibody as expected. The other fragment formed by HOCl at ~70 kDa, was not detected by the anti-His tag antibody indicating that this species does not contain the C-terminus. The immunostaining observed with the anti-His tag antibody did not increase in a dose-dependent manner with higher HOCl levels, possibly as a result of conformational modification of the binding domain of the antibody (His residues, a known target of HOCl [8,80]) by excess HOCl. In contrast, the anti-G1 domain antibody failed to recognize any of the fragments generated by HOCl. Although this may indicate that neither species contains the G1 epitope, this lack of signal may also reflect low sensitivity (poor recognition) or ready modification of the epitope recognized by the antibody. Some recognition of HOCl-mediated cross-linked species was however detected (Figure 3E) with this antibody under reducing (but not nonreducing) conditions, but at a low level when compared to that detected by silver staining or the anti-His tag antibody. Damage to the epitope is supported by the decreased antibody recognition of the parent protein at high HOCl excesses, and particularly under nonreducing conditions (Figure 3F). Thus, the origin of the 75 kDa fragment remains open.

The decrease in aggrecan cross-linking and fragmentation resulting from the presence of Met, when added before HOCl, is consistent with cleavage by this species. Addition of Met after 2 h had limited effects (Figure 4), suggesting that most modifications occur rapidly, and via either direct reaction of HOCl, or short-lived chloramines. Treatment with individual preformed chloramines indicates that these can induce fragmentation, with the extent of this process dependent on the chloramine. HEPES chloramines were inefficient (fragmentation only observed with ≥78-fold molar excess; see Figure 5), whereas Tau-CA and Lys-CA were moderately efficient (Figure 6 and Figure 7A), and His-CA highly efficient (Figure 7E). This pattern reflects the reactivity of these chloramines [7]. The less efficient detection of fragments by immunoblotting in these reactions when compared to silver staining (Figure 6 and Figure 7), is consistent with either a poor sensitivity of the antibodies, or significant epitope destruction.

rhAggrecan fragmentation and aggregation were also detected with chloramines formed on HSA, fibronectin, laminin, and basement membrane extracts. Although interpretation of the gels was complex due to the additional protein(s) present, strong evidence was obtained for similar processes to those seen with HOCl and low mass chloramines, and at low molar excesses (>10-fold) (Figure 9 and Figure 10). Fragments were also readily detected by Western blotting, suggesting that protein chloramines are inefficient at damaging the relevant epitopes.

In vivo, both aggrecan and ADAMTS1 are likely to be exposed to HOCl from MPO. The data obtained indicate that isolated ADAMTS1 is inactivated by HOCl in a dose-dependent manner (Figure 11). This inactivation was prevented by Met which scavenges HOCl. Unlike MMPs [58,66,67,68,69,70], no evidence was obtained for oxidant-mediated activation of ADAMTS1. This is likely to reflect differences in the structure of the ADAMTS and MMP families [81,82] and particularly the manner in which the activity of the zinc atom in the active site is controlled by the surrounding protein [83]. Thus the ADAMTS family lack the Cys residue present in the prodomain of MMPs that ligates to the zinc rendering the protein inactive; the ADAMTS family therefore cannot undergo the “cysteine-switch” mechanism that activates some MMPs [66].

When both aggrecan and ADAMTS1 were incubated concurrently with modest levels of HOCl, both oxidant mediated- and enzymatic cleavage were detected (c.f. the detection of both aggrecanase- and HOCl-induced fragments on the gels and membranes; Figure 12A,C,D). In contrast, at high oxidant levels enzymatic degradation was decreased (Figure 12C,D), consistent with ADAMTS1 inhibition, with mainly (or only) HOCl-mediated fragments detected. In contrast, with Tau-CA enzymatic fragments were detected at all but the highest oxidant excesses (Figure 12B), indicating that ADAMTS1 is resistant to inactivation by this chloramine. Inactivation of ADAMTS1 by HOCl, if this occurs in vivo, may result in a disturbance in aggrecan homeostasis, and accumulation during the development of atherosclerosis. Examination of human atherosclerotic plaques using an anti-aggrecan mAb confirmed that significant levels of aggrecan were present, in line with previous reports [50,51]. This proteoglycan is reported to impact adversely on smooth muscle cell viability via the induction of apoptosis [52], impact on the release of biologically active matrikines from aggrecan [45], and disrupt cell-ECM interactions. Determination of exact nature of the HOCl-induced fragments (using alternative approaches to the in-gel digestion/mass spectrometry approach tested here) and whether these fragments show matrikine activity, would be worthy of further study.

As smooth muscle cells are responsible for the synthesis of much of the ECM present in the fibrous caps of plaques, a decreased level of ECM synthesis may contribute to the correlation between high aggrecan levels and unstable plaques [52]. High aggrecan levels may also contribute to plaque instability by binding both MPO and LDL, thereby enhancing the extent of oxidation within plaques, and particularly of LDL, with consequent formation of lipid-laden foam cells and lipid cores. The generation of oxidants in proximity to aggrecan as a result of MPO binding would be expected to result in damage to aggrecan, as observed in the human atherosclerotic plaques examined here using mAb 2D10G9 that recognizes epitopes modified by HOCl, or a MPO-H_2_O_2_-Cl^−^ system, in vitro and in vivo [25,57]. Although this data does not provide definitive proof of HOCl-modified aggrecan in human atherosclerotic plaques, it is consistent with this hypothesis.

Overall, the data presented here indicate that both rhAggrecan and rhADAMTS1 are significant targets of HOCl and chloramines, with these reactions giving both aggregates and specific cleavage products. These reactions are facile and may be of significance in the development of human atherosclerotic plaques where elevated levels of both MPO, the source of HOCl and aggrecan are present. The observed fragmentation of aggrecan may adversely affect lesion development and stability by multiple mechanisms including loss of ECM integrity, altered aggrecan homeostasis and altered formation of biologically active matrikines.

## 5. Conclusions

The data presented here indicate that HOCl cleaves recombinant human aggrecan (consisting of the G1-IGD-G2 domains, but not the chondroitin sulfate binding sequence and G3 domains) at specific sites within the IGD domain. These sites of cleavage are different from those induced by the zinc-containing protease, ADAMTS1 which also cleaves within this region, and regulates aggrecan levels in vivo. Irreversible protein cross-links are also formed by HOCl in a dose-dependent manner. These effects are limited by the HOCl scavenger methionine. Chloramines present on amino acids, proteins, and extracellular matrix materials induce similar damage. Both HOCl and chloramines can inactivate ADAMTS1, suggesting that at sites of inflammation there may be a switch from proteolytic degradation to oxidative fragmentation of aggrecan. Evidence has also been obtained for colocalization of aggrecan and HOCl-generated epitopes in advanced human atherosclerotic plaques, though this does not provide definitive evidence for such damage. Together, these data show that HOCl and chloramines can induce specific modifications on aggrecan, and that these effects are distinct from those of ADAMTS1. Both processes may contribute to the accumulation of aggrecan fragments in ageing tissues.

## Data Availability

All data are available within the manuscript or on request from the corresponding authors.
